# Resting-state heart rate variability, level of stress and resilience in internet gaming disorder and alcohol use disorder

**DOI:** 10.3389/fphar.2023.1152819

**Published:** 2023-05-03

**Authors:** Jong Hu Park, So Young Yoo, Hye Yoon Park, Jung-Seok Choi

**Affiliations:** ^1^ Department of Psychiatry, Seoul National University Hospital, Seoul, South Korea; ^2^ Department of Psychiatry, SMG-SNU Boramae Medical Center, Seoul, South Korea; ^3^ Department of Psychiatry, Samsung Medical Center, Sungkyunkwan University School of Medicine, Seoul, South Korea

**Keywords:** internet gaming disorder (IGD), alcohol use disorder (AUD), heart rate variability (HRV), stress, resilience

## Abstract

Stress and resilience are involved in the pathophysiology of addictive disorders, and heart rate variability (HRV) is an index of an individual’s global capability to regulate psychological responses. In this study, we aimed to identify transdiagnostic and disorder-specific markers in people with addictive disorders by analyzing resting-state HRV and associations with the levels of stress and resilience. We compared relevant data between patients with internet gaming disorder (IGD) and/or alcohol use disorder (AUD) and healthy controls (HCs). In all, 163 adults aged 18–35 years (53 with IGD, 49 with AUD, 61 HCs) participated. The levels of stress and resilience were measured using the Psychosocial Wellbeing Index and the Connor-Davidson Resilience Scale, respectively. The HRV was obtained from each participant during a 5 min resting-state. The IGD and AUD patients exhibited increased levels of stress and decreased resilience compared to the HCs. Patients with either addictive disorder exhibited a lower standard deviation of the normal-to-normal beat interval (SDNN) index [SDNNi] compared to HCs even after adjusting for clinical variables such as depression, anxiety, and impulsivity. In multiple comparison tests among the three groups, the AUD group had lower HRV than HCs, but no differences were observed among the groups after adjusting for the clinical variables. The HRV indices were correlated with the levels of stress, resilience, and disease severity. In conclusion, IGD and AUD patients exhibit lower HRV as indicated by the SDNNi compared to HCs, revealing their vulnerability to stress as well as a common transdiagnostic marker of addiction.

## 1 Introduction

Internet gaming disorder (IGD) is a type of behavioral addiction characterized by playing games excessively so that it interferes with social relationships and work performance. As the internet is available to most people, and communication through the internet has become common among young people, IGD is emerging as a new social issue. The Diagnostic and Statistical Manual of Mental Disorders-Fifth Edition (DSM-5) includes IGD as a condition proposed for future study, whereas the 11th revision of the International Classification of Diseases (ICD-11) added gaming disorder as a mental disorder. The diagnostic criteria consist of impaired control over gaming, negative psychological problems, and impaired social or occupational functioning. The global prevalence of gaming disorder is estimated to be 3.05% (Stevens et al., 2021). The prevalence of IGD varies widely from 1.16% reported in Germany (Rehbein et al., 2015) to 17.0% reported in China (Liao et al., 2020). In Korea, the prevalence is 5.9% (10.4% in boys and 1.2% in girls) in middle-school students (Yu and Cho, 2016) and 10.8% of people aged 14–39 years (Wang et al., 2018). IGD is associated with psychiatric symptoms, such as depression and anxiety, and other mental diseases, such as mood disorders (Kim et al., 2016). Young people have spent more time at home recently, and IGD appears to have been aggravated by the COVID-19 pandemic. Probable prevalence of IGD in Japan during the pandemic seems to have increased by more than 1.6 times compared to before the pandemic, rising from 3.7% to 5.9% (Oka et al., 2021). Behavioral addictions, such as gaming, food, or gambling addictions, share some features with substance addictions including impaired control over engagement, continuous engagement despite harmful consequences, and urges or cravings (Zhang et al., 2017). Adequate online gaming or social gambling activities to reduce negative feelings and communicate with others can be culturally accepted forms of entertainment (Griffiths, 2019; Dong et al., 2020) and become problematic or pathological among vulnerable subjects who face psychosocial or environmental distress (Maniaci et al., 2015). If addictive behaviors develop, it is common for patients with an addictive disorder to develop cross-addictive behaviors, and network analysis has shown that addictive behaviors are connected to other types of addictions (Zarate et al., 2022).

The association between stress and addictive disorders has been suggested by several studies. Stress acts as a challenge to brain circuit regulatory mechanisms, such as the corticotropin-releasing factor system, and promotes allostatic modulation resulting in addiction, an allostatic state (Koob and Schulkin, 2019). Resilience is the ability to cope with stress and adversity (Sarkar and Fletcher, 2014). Individuals with higher resilience have better mental wellbeing and are less vulnerable to psychiatric disorders (Shrivastava and Desousa, 2016). In previous studies on the role of resilience in patients with IGD, the IGD severity score was associated with high perceived stress and low resilience (Canale et al., 2019), and IGD patients with lower or moderate resilience levels exhibit more depressive symptoms and higher stress levels (Lee et al., 2019).

Heart rate variability (HRV) is the variation in the time interval between consecutive heartbeats and is measured by analyzing the R-R intervals in echocardiographic data. HRV is an indicator of autonomic nervous system activity (Jung et al., 2019). The analysis of HRV consists of time-domain and frequency-domain analyses. HRV is related to cerebral blood flow in several regions involved in the response to stress (Williams et al., 2019) and is an index of an individual’s global capability to regulate psychological responses adaptively when facing stress (Perna et al., 2020). In particular, resting state HRV has been found to be associated with chronic stress (Jiryis et al., 2022) as well as many psychiatric disorders. Several studies have demonstrated an association between HRV and psychiatric disorders, such as depression (Agelink et al., 2002) and post-traumatic stress disorder (Lee and Theus, 2012). HRV is also known to be related to addictive disorders. In a meta-analysis (Cheng et al., 2019) of alcohol use disorder (AUD) patients, HRV indices were lower in addicted patients. In another study, problematic internet users had low measured baseline SDNN values (Moretta and Buodo, 2018).

In this study, we measured the resting state HRV in both IGD and AUD patients and assessed how it reflects stress and resilience in patients. By recruiting patient groups of both IGD, a type of behavioral addiction, and AUD, at type of substance addiction, we aimed to identify the specific characteristics of IGD as a behavioral addiction by comparing to AUD and HC. This study hypothesizes that IGD patients have low HRV during rest as well as decreased resilience and are more susceptible to stress than healthy controls (HCs). Furthermore, both groups of patients would have common transdiagnostic and disorder-specific HRV markers.

## 2 Methods

### 2.1 Participants

In all, 163 adults (aged 18–35 years) were recruited from SMG-SNU Boramae Medical Center and the local community in Seoul, South Korea. SMG-SNU Boramae Medical Center is a public hospital in Seoul that treats patients from diverse psychiatric backgrounds and operates an addiction center. The patients with IGD and AUD were seeking treatment for their addiction and were diagnosed by a clinically experienced psychiatrist according to DSM-5 criteria. Fifty-three of the participants were diagnosed with IGD, 49 were diagnosed with AUD, and 61 were HCs. Individuals who had a history of comorbid psychiatric disorders, significant head injury, or cognitive delay were excluded. All participants were medication-naïve at the time of the assessment.

The IGD patients spent more than 4 h per day and 30 h per week playing internet games, and consumed fewer than 14 standard drinks per week. The AUD patients played games for less than 2 h per day and had abstained from alcohol use for at least 2 weeks before participating in the study, which was verified by self-reports and reports from caregivers. The severity of the addictive disorder was assessed using Young’s Internet Addiction Test (Y-IAT) in IGD patients and the Alcohol Use Disorder Identification Test (AUDIT) in AUD patients.

Adults from the local community who played games for less than 2 h per day and drank fewer than 14 standard drinks per week were recruited for the HC group through advertisements. None had a history of any psychiatric disorder.

This study protocol adhered to the principles of the Declaration of Helsinki and was approved by the Institutional Review Board of SMG-SNU Boramae Medical Center. All participants understood the study procedure and provided written informed consent before participation.

### 2.2 Measures

#### 2.2.1 Young’s internet addiction test

The Y-IAT was used to assess IGD severity. It includes 20 items rated on a 5-point scale. The total score ranges from 20 to 100, and total scores were calculated using Young’s method (Young, 1998; Lee et al., 2013). The items of Y-IAT assess characteristics related to internet use such as escapism, compulsivity, dependency, and social or occupational functioning. Total scores ranging from 31 to 49 indicate mild internet addiction, scores of 50–79 indicate moderate addiction, and scores of 80 or above suggest severe addiction (Samaha et al., 2018). In this study, all patients in the IGD group exhibited Y-IAT scores above 30, with an average score of 63.66. The Cronbach’s alpha coefficient was 0.96.

#### 2.2.2 Alcohol use disorder identification test-Korean version

The Korean version of the AUDIT (Kim et al., 1991) was used to evaluate the severity of AUD in this study. It consists of 10 items on a 4-point scale measuring the frequency of alcohol abuse behavior. Higher scores reflect greater severity of AUD symptoms. The AUDIT scores of 8 or more are suggested as indicators of hazardous and harmful alcohol use (Babor et al., 2001), and the lowest AUDIT score in the AUD patient group in this study was 11. The mean AUDIT score of the AUD patients was 26.06. The Cronbach’s alpha coefficient was 0.92.

#### 2.2.3 Beck Depression Inventory-II (BDI)

The BDI (Beck et al., 1996; Lim et al., 2011) is a self-reporting instrument consisting of 21 items and including four statements measuring the severity of particular symptoms experienced over the past week. It was used to assess the severity of depression symptoms. The Cronbach’s alpha coefficient was 0.98.

#### 2.2.4 Beck Anxiety Inventory (BAI)

The BAI (Beck et al., 1988; Kwon, 1997) is a self-reporting instrument that consists of 21 items rated over the past week on a 4-point scale. It was used to measure the severity of anxiety symptoms. The Cronbach’s alpha was 0.96.

#### 2.2.5 Barratt Impulsiveness Scale-11 (BIS-11)

The severity of impulsivity was measured with the BIS-11 (Barratt, 1985), which includes 11 items on a 4-point Likert scale, including the subscales of cognitive impulsiveness, motor impulsiveness, and non-planning impulsiveness. The Cronbach’s alpha was 0.86.

#### 2.2.6 Connor-Davidson Resilience Scale (CD-RISC)

The CD-RISC (Connor and Davidson, 2003) is a self-reporting instrument consisting of 25 items on a 5-point Likert scale. The CD-RISC was used to evaluate the level of resilience. This scale asks participants how they felt over the past month. The Cronbach’s alpha coefficient was 0.96.

#### 2.2.7 Psychosocial Wellbeing index (PWI)

The PWI (Kim, 1999) is a self-reporting scale modified to meet the characteristics of the Korean population. It is composed of 45 items used to assess psychological stability by evaluating social role performance, self-confidence, depression, sleep disturbance, anxiety, and general wellbeing. Each item evaluates psychological status, such as performance of social roles, self-confidence, depression, sleep disturbance, anxiety, and general wellbeing over the prior few years. Higher scores reflect greater levels of distress. The Cronbach’s alpha coefficient was 0.91.

### 2.3 Heart rate and HRV

#### 2.3.1 ECG data

The acquisition of the heart rate and HRV data was conducted before the administration of the questionnaires in a quiet environment and in a relaxed, seated position. ECG data were obtained for 5 min from each participant by placing Ag/AgCl electrodes on the left and right supraclavicular areas. All participants were required to restrict caffeine and nicotine use for 2 h before the measurements.

#### 2.3.2 HRV analysis

After collecting the ECG data, we used the Pan and Tomkins algorithm to obtain all R-R intervals automatically. We preprocessed the R-R interval data to remove ectopic beats by applying a 20% filter and interpolating with 4 Hz. Then we performed a power spectrum analysis of the ECG signal using HRV analysis software in the MATLAB environment (Math Works, Natick, MA, USA).

We calculated the mean heart rate (HR) and analyzed the HRV data in the time and frequency domains. In time-domain analysis, HRV indices, such as the standard deviation of the normal to normal [R-R] beat interval (SDNN), the average of the SDNN for each 50-s segment (SDNN index [SDNNi]) reflecting short-term HRV variability, the root mean square of successive R-R differences (RMSSD), and the percentage of successive R-R differences by more than 50 m (pNN50) are frequently used (Jung et al., 2019; Perna et al., 2020). In the frequency domain analysis, we used power spectrum analysis to analyze the HRV data in specific frequency bands, including very low frequency (VLF, 0.005–0.04 Hz), low frequency (LF, 0.04–0.15 Hz), and high frequency (HF, 0.15–0.4 Hz). The low-frequency (0.04–0.15 Hz) component is associated with the sympathetic activity of the heart, and the high-frequency component reflects vagal modulation (Jung et al., 2019). We also used the logarithmically transformed value of each frequency band.

### 2.4 Statistical analysis

The Shapiro-Wilk test was conducted to test the normality of the data distribution. Analysis of variance was performed to compare the mean values of the demographic data and the clinical parameters among the three groups (IGD, AUD, and HC). The multiple comparison method was adopted when the difference among the groups was significant. We also conducted a comparison between the addictive disorder groups (IGD and AUD) and the HC group using the *t*-test to explain common characteristics of addictive disorders and to identify transdiagnostic markers for addictive disorders. We conducted analysis of covariance for the differences in the HRV indices between the groups after adjusting for the clinical variables (BDI, BAI, and BIS-11). Pearson’s correlation analysis was performed to examine the relationships between the HRV indices and the stress-related variables (PWI and CD-RISC) in each group and to evaluate the relationships between the HRV indices and the disease severity indices (AUDIT for AUD and Y-IAT for IGD). *p*-values <0.05 were considered significant for all analyses.

## 3 Results

### 3.1 Demographic and clinical characteristics

The demographic and clinical characteristics of the three groups are described in Table 1. The mean age of all participants was 25.29 years. The mean age of the AUD group was older than the HC group, and the mean age of the IGD group was younger than the HC group. Most of the participants were male, and the proportion of females was highest in the AUD group. Patients from both groups showed higher BDI, BAI, and BIS-11 scores than those in the HC group.

**TABLE 1:** Demographic and clinical characteristics.

	IGD	AUD	HC	*p*
n	53	49	61	
SEX	M:50, F:3	M:38, F:11	M:52, F:9	0.120^a^
**AGE**	**23.66 ± 5.02**	**27.51 ± 5.01**	25 ± 3.37	<0.001*
**AUDIT**	5.3 ± 4.91	**26.06 ± 6.77**	4.58 ± 3.26	<0.001*
**Y_IAT**	**63.66 ± 15.27**	34.31 ± 14.45	30.03 ± 8.85	<0.001*
**BDI**	**18.7 ± 11.84**	**22.47 ± 15.49**	4.07 ± 4.08	<0.001*
**BAI**	**16.23 ± 13.45**	**19.94 ± 15.25**	4.92 ± 5.32	<0.001*
**BIS-11**	**66.66 ± 10.24**	**69.16 ± 10.92**	56.36 ± 7.66	<0.001*

^**a**^ Chi-square test, *p < .05. Data values of statistical significance are expressed in bold characters.

IGD: internet gaming disorder; AUD: alcohol use disorder; HC: healthy control; AUDIT: alcohol use disorder identification test; Y-IAT: Young’s Internet Addiction Test; BDI: Beck Depression Inventory-II; BAI: beck anxiety inventory; BIS-11: Barratt Impulsiveness Scale-11.

### 3.2 Stress-related variables (PWI and resilience) in addictive disorders and HCs

**FIGURE 1:**
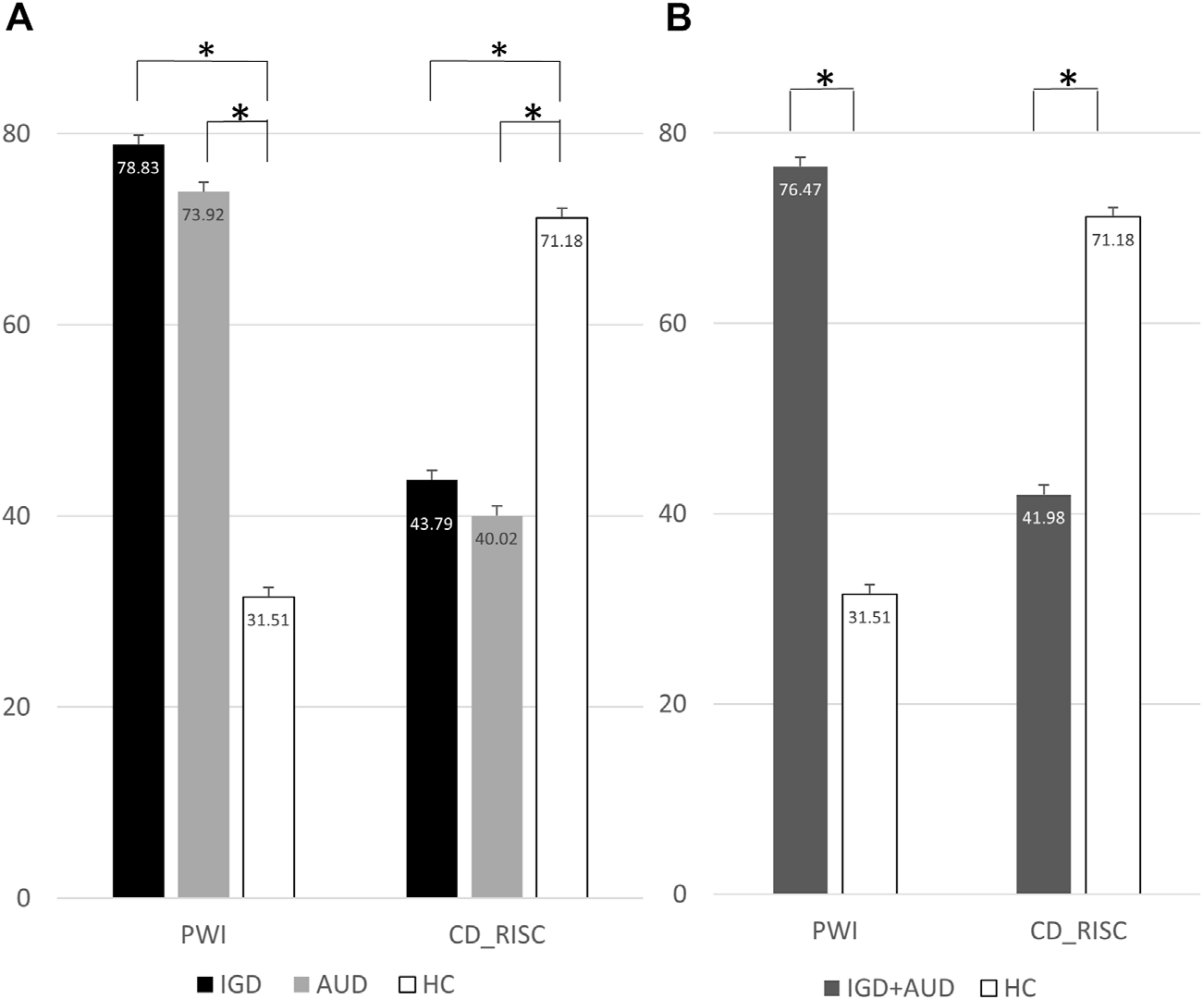
**(A)** Differences of PWI and CD-RISC in 3 groups (IGD, AUD, and HC). **(B)** Differences of PWI and CD-RISC in 2 groups (IGD + AUD and HC). Each bar presented mean and standard error (SE). *p < .05. IGD, Internet gaming disorder; AUD, Alcohol use disorder; HC, Healthy control; PWI, Psychosocial wellbeing index; CD-RISC, Connor-Davidson resilience scale.

The IGD and AUD groups exhibited higher mean PWI scores than the HC group. The mean CD-RISC scores were significantly lower in the AUD and IGD groups than in the HC group (Figure 1A). The addictive disorder groups (IGD and AUC) had a higher level of stress and a lower level of resilience than the HC group (Figure 1B).

### 3.3 HRV indices in the three groups

The mean HR was higher in the IGD and AUD groups than in the HC group. The logarithmically transformed value of the low-frequency band (Log_LF) and the SDNNi were lower in the AUD group than in the HC group (Table 2). However, after adjusting for the clinical variables, such as the BDI, BAI, and BIS-11, no significant differences were detected among the groups.

**TABLE 2:** Heart rate variability indices among IGD, AUD, and HC.

		IGD	AUD	HC	*p*
**HR**	**Unadjusted**	73.95 ± 11.24*	73.44 ± 10.54*	**67.69 ± 9.9**	**0.002**
	Adjusted	73.47 ± 1.5	72.64 ± 1.66	68.74 ± 1.61	0.126
**Frequency Domain**					
LF	Unadjusted	544.32 ± 816.48	394.81 ± 581.38	502.9 ± 477.26	0.475
	Adjusted	554.73 ± 89.51	421.82 ± 98.81	472.16 ± 95.88	0.561
HF	Unadjusted	121.19 ± 87.62	104.9 ± 77.65	109.01 ± 51.66	0.496
	Adjusted	124.45 ± 10.36	110.52 ± 11.44	101.67 ± 11.1	0.331
**Log_LF**	**Unadjusted**	**5.73 ± 1.06**	5.31 ± 1.22*	**5.8 ± 0.96**	**0.045**
	Adjusted	5.76 ± 0.15	5.39 ± 0.17	5.71 ± 0.16	0.206
Log_HF	Unadjusted	4.51 ± 0.84	4.36 ± 0.86	4.57 ± 0.53	0.344
	Adjusted	4.57 ± 0.1	4.47 ± 0.12	4.43 ± 0.11	0.635
**Time Domain**					
SDNN	Unadjusted	39.73 ± 19.44	34.37 ± 17.72	41.59 ± 18.17	0.117
	Adjusted	40.14 ± 2.62	35.3 ± 2.9	40.48 ± 2.81	0.364
**SDNNi**	**Unadjusted**	**389.33 ± 112.81**	378.81 ± 99.91*	**433.69 ± 108.27**	**0.017**
	Adjusted	388.61 ± 15.34	378.07 ± 16.93	434.9 ± 16.43	0.074
RMSSD	Unadjusted	41.61 ± 28.39	35.97 ± 21.26	43.8 ± 23.2	0.239
	Adjusted	41.93 ± 3.46	36.79 ± 3.82	42.86 ± 3.71	0.490
pNN50	Unadjusted	69.67 ± 16.86	68.95 ± 17.78	73.63 ± 13.18	0.243
	Adjusted	70.91 ± 2.24	71.29 ± 2.47	70.67 ± 2.39	0.986

*p < .05, compared to healthy control. Data values of statistical significance are expressed in bold characters.

IGD: internet gaming disorder; AUD: alcohol use disorder; HC: healthy control; HR: mean heart rate; LF: Low-frequency band; HF: High-frequency band; Log_LF: Logarthmically transformed value of low-frequency band; Log_HF: Logarthmically transformed value of high-frequency band; SDNN: The standard deviation of normal-to-normal [R-R] intervals; SDNNi: SDNN index, the average of SDNN, for each segment of 50 s length; RMSSD: The root mean square of successive R-R interval differences; pNN50: The percentage of successive R−R intervals differing more than 50 milliseconds.

### 3.4 HRV indices between disorder groups and HCs

Next, two-group analysis combining the IGD and AUD groups as one addictive disorder group was performed. The mean HR was higher in the disorder group than in the HC group. SDNNi was significantly lower in the disorder group (Table 3), even after adjusting for the clinical variables.

**TABLE 3:** Heart rate variability indices in addictive disorder and HC.

		IGD + AUD	HC	*p*
**HR**	**Unadjusted**	**73.7 ± 10.86**	**67.69 ± 9.9**	0.001*
	**Adjusted**	**73.1 ± 1.16**	**68.7 ± 1.6**	0.045*
**Frequency Domain**				
LF	Unadjusted	472.5 ± 713.79	502.9 ± 477.26	0.768
	Adjusted	495.55 ± 69.58	464.36 ± 95.62	0.819
HF	Unadjusted	113.37 ± 82.98	109.01 ± 51.66	0.712
	Adjusted	118.24 ± 8.05	100.86 ± 11.06	0.252
Log_LF	Unadjusted	5.53 ± 1.15	5.8 ± 0.96	0.118
	Adjusted	5.59 ± 0.12	5.69 ± 0.16	0.659
Log_HF	Unadjusted	4.44 ± 0.85	4.57 ± 0.53	0.291
	Adjusted	4.53 ± 0.08	4.43 ± 0.11	0.515
**Time Domain**				
SDNN	Unadjusted	37.15 ± 18.74	41.59 ± 18.17	0.141
	Adjusted	37.98 ± 2.04	40.2 ± 2.81	0.565
**SDNNi**	**Unadjusted**	**384.28 ± 106.41**	**433.69 ± 108.27**	0.005*
	**Adjusted**	**383.92 ± 11.89**	**434.29 ± 16.34**	0.026*
RMSSD	Unadjusted	38.9 ± 25.25	43.8 ± 23.2	0.218
	Adjusted	39.64 ± 2.69	42.56 ± 3.7	0.565
pNN50	Unadjusted	69.32 ± 17.23	73.63 ± 13.18	0.095
	Adjusted	71.08 ± 1.73	70.69 ± 2.38	0.904

*p < .05. Data values of statistical significance are expressed in bold characters.

IGD: internet gaming disorder; AUD: alcohol use disorder; HC: healthy control; HR: mean heart rate; LF: Low-frequency band; HF: High-frequency band; Log_LF: Logarthmically transformed value of low-frequency band; Log_HF: Logarthmically transformed value of high-frequency band; SDNN: The standard deviation of normal-to-normal [R-R] intervals; SDNNi: SDNN index, the average of SDNN, for each segment of 50 s length; RMSSD: The root mean square of successive R-R interval differences; pNN50: The percentage of successive R−R intervals differing more than 50 milliseconds.

### 3.5 Correlational analysis

**FIGURE 2:**
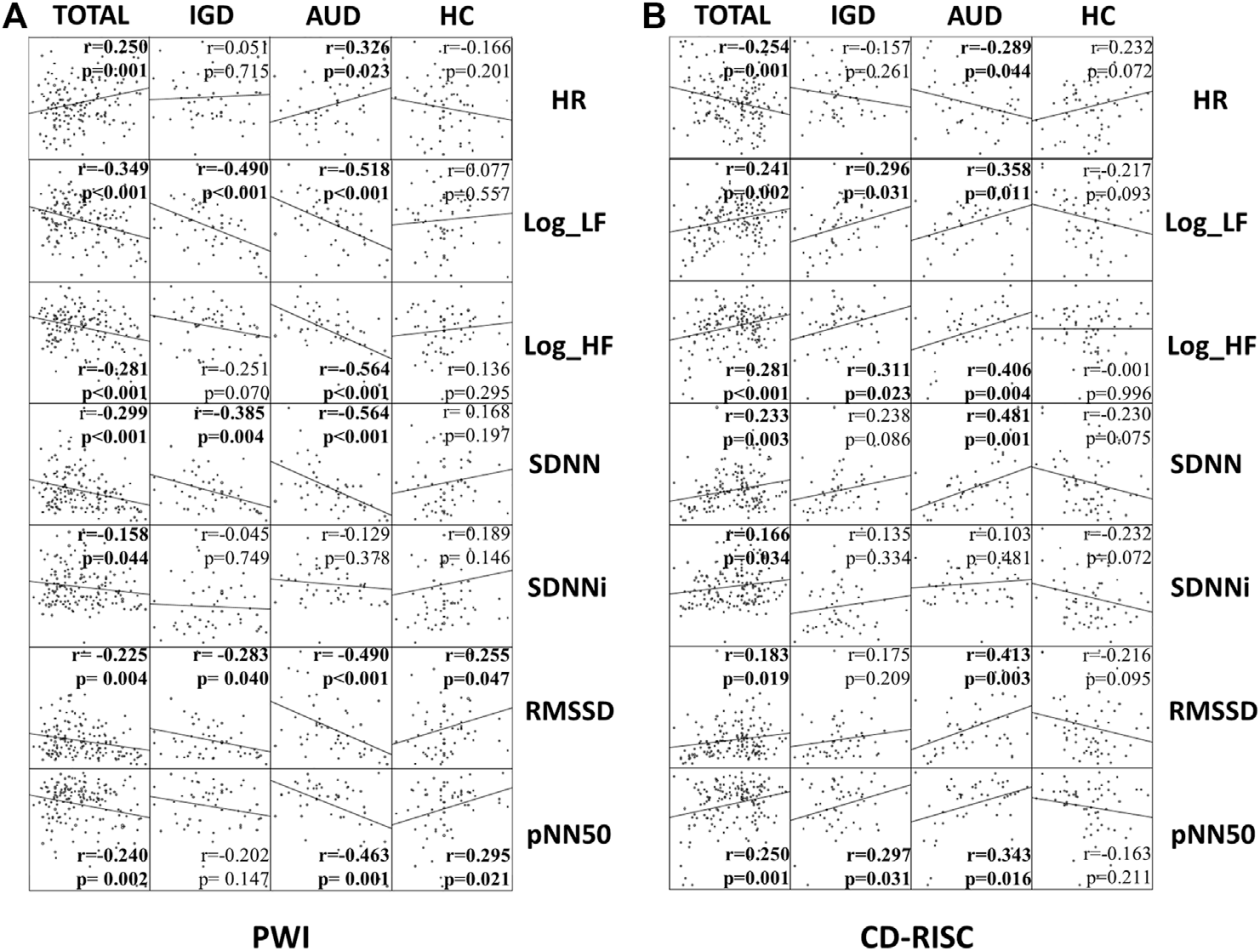
**(A)** Correlational analysis: PWI (Stress) and HRV indices. **(B)** Correlational analysis: CD-RISC (Resilience) and HRV indices. The correlation coefficient and *p*-value in each group (all participants (TOTAL), IGD, AUD, and HC) are specified in each plot. IGD**,** Internet gaming disorder; AUD**,** Alcohol use disorder; HC**,** Healthy control; PWI**,** Psychosocial wellbeing index; CD-RISC**,** Connor-Davidson resilience scale; HR**,** Mean heart rate; Log_LF**,** Logarthmically transformed value of low-frequency band; Log_HF**,** Logarthmically transformed value of high-frequency band; SDNN**,** The standard deviation of normal-to-normal [R-R] intervals; SDNNi**,** SDNN index, the average of SDNN for each segment of 50 s length; RMSSD**,** The root mean square of successive R-R interval differences; pNN50**,** The percentage of successive R−R intervals differing more than 50 milliseconds.

We conducted a correlation analysis of the PWI, which measures the level of stress, and the HRV indices. Log_LF, SDNN, and the RMSSD in the IGD and AUD groups were negatively correlated with the PWI. HR, logarithmically transformed value of the high-frequency band (Log_HF), and pNN50 were significantly correlated with the PWI only in the AUD group (Figure 2A). The HRV indices Log_LF, Log_HF, and pNN50 were positively correlated with the CD-RISC in the IGD and AUD groups. HR, SDNN, and RMSSD were correlated with CD-RISC only in the AUD group (Figure 2B).

**FIGURE 3:**
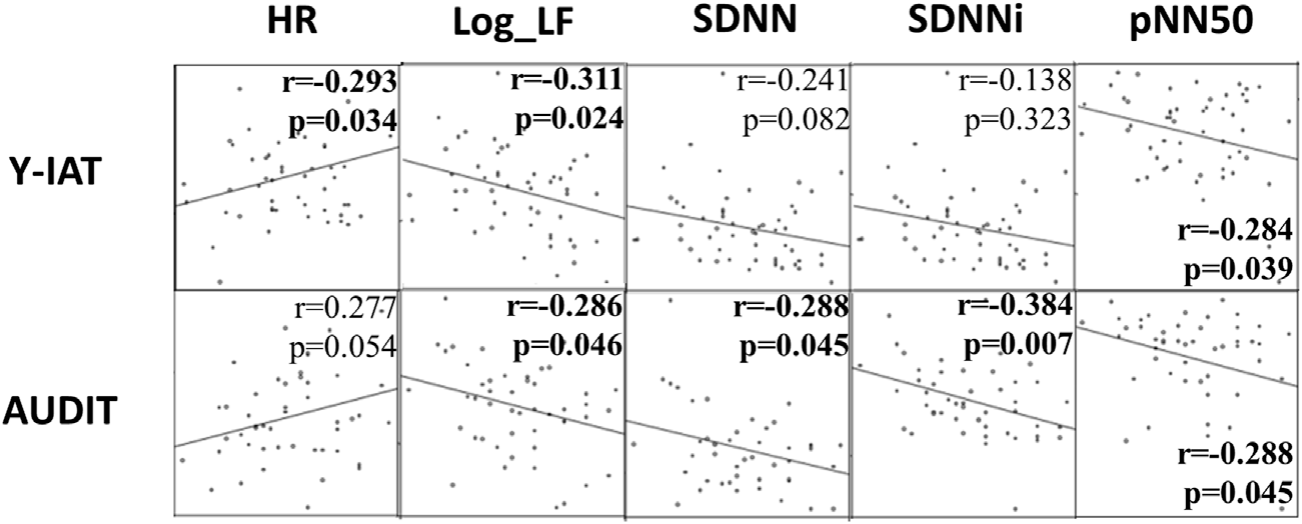
Correlational analysis: Y-IAT and AUDIT with HRV indices. The correlation coefficient and *p*-value are specified in each plot. Y-IAT, Young’s Internet Addiction Test; AUDIT, Alcohol Use Disorder Identification Test; HR, Mean heart rate; Log_LF, Logarthmically transformed value of low-frequency band; SDNN, The standard deviation of normal-to-normal [R-R] intervals; SDNNi, SDNN index, the average of SDNN for each segment of 50 s length; pNN50, The percentage of successive R−R intervals differing.

The HRV indices Log_LF and pNN50 were correlated with the Y-IAT for IGD and the AUDIT for AUD. SDNN and SDNNi were correlated with the AUDIT. HR was correlated with the Y-IAT (Figure 3).

## 4 Discussion

This study compared an IGD group as an example of behavioral addiction with an AUD group as an example of substance addiction. It evaluated the stress level and resilience in each group and how they were reflected in the HRV indices. The study aimed to investigate the distinctive features of each group by conducting inter-group comparisons while minimizing the influence of potential confounding variables. To achieve this objective, we excluded individuals with prior psychiatric histories during participant recruitment and imposed limits on alcohol consumption for individuals in the IGD group and on gaming hours for those in the AUD group. Furthermore, a 2-week cessation period was introduced to mitigate the impact of alcohol on the autonomic nervous system. Additionally, we measured the BDI, BAI, and BIS-11 in every participant. Impulsivity is a significant factor in the early stage of addiction (Zhang et al., 2017). Anxiety and depression are associated with a higher risk of developing both addictive disorders, IGD (Kim et al., 2016) and AUD (Boschloo et al., 2011). Therefore, in our study, we attempted to identify disorder-specific markers by analyzing measurements which were adjusted for clinical variables such as depression, anxiety, and impulsivity. We also performed additional analysis by merging the two disorder groups to determine what HRV indices may be useful as common transdiagnostic markers for addictive disorders.

In the two-group analysis that compared the combined disorder group with the HC group, the PWI score was higher and the CD-RISC score was lower in the disorder group. This finding is consistent with previous studies that patients with addictive disorders are vulnerable to stress due to low resilience (Canale et al., 2019; Lee et al., 2019) and that stress affects the regulatory mechanism of the brain acting as an allostatic load that causes addiction (Koob and Schulkin, 2019).

The dopamine circuit plays a crucial role in the pathophysiology of addiction disorders, and individual differences in dopamine system activity and function are important factors in addiction. Striatal dopamine has been associated not only with positive symptoms of substance abuse such as craving, but also with compulsive behavior, reduced sociality, and risk-taking (Poisson et al., 2021). In Parkinson’s disease, a neurodegenerative condition characterized by dopaminergic neuronal loss, research has shown that the degree of neuronal loss is associated with changes in HRV (Warhadpande et al., 2022). Another study has found that striatal dopamine activity correlates with parasympathetic modulation reflected by HRV (Yeh et al., 2006). Moreover, a previous study that measured both HRV and electroencephalography (EEG) data in patients with IGD found that the patients exhibited heightened theta band characteristic path lengths on EEG compared to healthy controls, and that these were negatively correlated with the SDNNi value (Park et al., 2020). These findings suggest that changes in the dopamine circuit system in addicted patients affect autonomic regulation by the brain and heart, and that the results from HRV analysis may help explain the pathophysiology of addictive disorders.

In our study, patients with an addictive disorder tended to have higher mean HRs and lower measured SDNNi values than those of healthy participants. The difference in SDNNi remained significant even after adjusting for clinical variables, such as the BDI, BAI, and BIS-11, which are clinical indicators of depression, anxiety, and impulsiveness, respectively. Therefore, SDNNi is suggested to be a transdiagnostic marker for addictive disorders. Log-LF and pNN50 were significantly correlated with both disorders in the correlation analysis with Y-IAT and AUDIT. These indices can be used as markers reflecting the severity of an addictive disorder.

No significantly different HRV indices were detected between the IGD and AUD groups. The HRV indices in the IGD group were not significantly different from those in the HC group. The AUD group had lower Log-LF and SDNNi values than those in the HC group. However, when the clinical variables were adjusted, no significant differences were observed between the AUD and HC groups, indicating that the lower Log-LF and SDNNi values in patients with AUD may be associated with mood states and impulsivity. More HRV indices were correlated with stress levels, resilience, and disease severity in the AUD group than in the IGD group. According to these results, the use of HRV indices as a disorder-specific marker is likely to be more plausible in patients with AUD than in those with IGD, but further research is needed on the effects of clinical variables such as depression, anxiety, and impulsivity.

We can suggest some reasons to explain why there were differences between the IGD group and the AUD group. First, AUD patients were older than the IGD patients in this study, and thus have had longer periods of morbidity. Therefore, the brain system of AUD patients had been on allostatic modulation for a longer period, and there was more of an influence on the brain-heart interaction in them. Second, unlike IGD, which is a behavioral addiction, in AUD, alcohol enters the patient’s body and affects various organs, including the brain. Thus, alcohol acts directly on the central nervous system. In addition, although it was not a significant difference, the proportion of women in the AUD group tended to be higher and the sex difference should have been considered. We also conducted additional analysis using only male participants. The overall trend in the difference among groups remained similar to the previous analysis, but the differences in SDNNi in two group analysis with adjusted data became statistically insignificant. To determine whether these findings are due to different physiological characteristics of women or decreased statistical power resulting from a smaller sample size, a future study should include an analysis that separates men and women in a larger sample.

The mean age of each group was in their 20s, with most participants being in early adulthood. Addictive disorders usually onset during adolescence or early adulthood, with 80% of alcoholism occurring before the age of 30 (Helzer et al., 1991; Chambers et al., 2003), and gaming addiction is known to be highly prevalent in adolescence (Festl et al., 2013). Adolescence and early adulthood are periods of high importance when neurodevelopment continues in brain regions associated with motivation, impulsivity, and addiction. The early onset of substance use has been linked to psychosocial issues in individuals with substance use disorders (Poudel and Gautam, 2017). Moreover, starting to play games at a young age has been identified as a significant predictor of problematic gaming (Mentzoni et al., 2011; Jeong et al., 2021). Therefore, it is necessary to analyze the characteristics of individuals with addictive disorders during these periods and study early intervention methods.

The significance of this study is that it analyzed IGD as a behavioral addiction and AUD as a substance addiction together. In addition, we excluded the influence of medication because we evaluated only drug-naive participants.

Some limitations of this study should be discussed. First, selection bias may have occurred when recruiting the patients and HCs. The mean age was significantly older in the AUD group and younger in the IGD group than in the HC group. We did not consider the difference in socioeconomic status during recruiting. Second, there was no common indicator of disease severity in the two addictive disorders. Therefore, there may be a difference in addiction severity between the IGD group and the AUD group. Finally, we obtained ECG data only in the resting state; thus, we have limited results to evaluate the direct response to the stressful situation and the recovery process.

## 5 Conclusion

IGD and AUD patients showed higher stress levels and lower resilience than HCs. The patients exhibited lower HRV, as indicated by the SDNNi, compared to HCs, revealing their vulnerability to stress. The HRV indices were correlated with stress levels, resilience, and disease severity. More HRV indices were correlated in the AUD group than in the IGD group. We found no disease-specific marker for IGD. Further research is needed on HRV indices and their relationships with clinical variables such as depression, anxiety, and impulsivity.

Alterations in the brain-heart system as reflected in HRV were observed in patients in their early 20s, the critical period in the pathophysiology of addictive disorders. This highlights the importance of examining the features of young patients with addiction for the purposes of prevention and early intervention.

## Data Availability

The raw data supporting the conclusions of this article will be made available by the authors, without undue reservation.
